# Association between polymorphisms and hypermethylation of *CD36* gene in obese and obese diabetic Senegalese females

**DOI:** 10.1186/s13098-022-00881-2

**Published:** 2022-08-18

**Authors:** Maïmouna Touré, Aziz Hichami, Amira Sayed, Muhtadi Suliman, Abdoulaye Samb, Naim Akhtar Khan

**Affiliations:** 1 Laboratoire de Physiologie Humaine et d’Explorations Fonctionnelles, Faculté de Médecine, de Pharmacie et d’Odonto-Stomatologie (FMPOS) de l’Université Cheikh Anta Diop (UCAD), Dakar, Sénégal; 2 Physiologie de La Nutrition & Toxicologie, INSERM U1231, Université de Bourgogne-Franche Comté (UBFC), Dijon AgroSup, 21000 Dijon, France; 3 IRL3189 ESS (Environnement, Santé, Sociétés ), CNRS, CNRST, Bamoko-UCAD, Dakar, Sénégal

**Keywords:** Obesity, Type 2 diabetes, *CD36* gene, Polymorphisms, Methylation

## Abstract

**Background:**

Obesity and related metabolic disorders are associated with genetic and epigenetic alterations. In this study, we have examined the association between polymorphisms and hypermethylation of the *CD36* gene promoter with obesity in Senegalese females with or without type 2 diabetes mellitus to identify novel molecular markers of these pathologies (obesity and type 2 diabetes mellitus).

**Materials and methods:**

The study was conducted in Senegal with healthy lean control, obese, and obese diabetic (age; 49.98 years ± 7.52 vs 50.50 years ± 8.76 vs 51.06 ± 5.78, and body mass index (BMI); 24.19 kg/m^2^ ± 2.74 vs 34.30 kg/m^2^ ± 4.41 vs 33.09 kg/m^2^ ± 4.30). We determined three genetic polymorphisms of *CD36* i.e., rs1761667, rs1527483, and rs3211867 by real-time polymerase chain reaction, and methylation of CPG islands of *CD36* was assessed by methylation-specific polymerase chain reaction (MS-PCR) in DNA isolated from peripheral blood of each participant. Plasma sCD36 levels and DNA methyltransferase 3a (DNMT3a) levels were determined by enzyme-linked immunosorbent assay (ELISA). According to the standard laboratory protocol, all biochemical parameters were analyzed from fasting serum or plasma.

**Results:**

For rs1761667, obese and obese diabetic subjects had statistically significant different parameters depending on the genotypic distribution. These were waist size for obese and HDL cholesterol for obese diabetic, they were significantly higher in subjects harboring GG genotype of rs1761667 (respectively p = 0.04 and p = 0.04).

For rs3211867, obese subjects harboring the AA/AC genotype had significantly higher BMI (p = 0.02) and total cholesterol (p = 0.03) than obese subjects harboring the CC genotype. At the same time, the obese diabetic subjects harboring the AA/AC genotype had total cholesterol levels significantly higher than the obese diabetic subjects harboring the CC genotype (p = 0.03). For rs1527483, only the control subjects had statistically significant different parameters depending on the genotypic distribution. The control subjects harboring the GG genotype had a significantly higher BMI than the control subjects harboring the AA/AG genotype (p = 0.003). The *CD36* gene methylation was significantly 1.36 times more frequent in obese and obese diabetic compared to lean control (RR = 1.36; p = 0.04). DNMT3a levels were higher in subjects with *CD36* gene methylation than in subjects without *CD36* gene methylation in each group. Obese diabetic subjects with *CD36* gene methylation had significantly fewer plasmas sCD36 (p = 0.03) and more LDL-cholesterol (p = 0.01) than obese diabetic subjects without *CD36* gene methylation. In the control group, an increase in sCD36 levels would be associated with a decrease in total cholesterol and triglyceride levels (coef = -7647.56 p = 0.01 and coef = -2528.50 p = 0.048, respectively) would be associated with an increase in LDL cholesterol levels. For the obese group, an increase in sCD36 levels would be associated with an increase in fasting insulin levels (coef = 490.99 p = 0.02) and a decrease in glycated hemoglobin levels (coef = -1196.26 p = 0.03). An increase in the sCD36 levels would be associated with an increase in the triglyceride levels in the obese diabetic group (coef = 9937.41 p = 0.02). The AA/AC genotype of SNP rs3211867 polymorphism was significantly associated with *CD36* gene methylation in the control and obese diabetic groups (respectively p = 0.05, p = 0.002; 95% CI).

**Conclusion:**

These observations suggest that polymorphisms and epigenetic changes in *CD36* gene promoters may be implicated in the onset of obesity and its related complication type 2 diabetes mellitus.

**Supplementary Information:**

The online version contains supplementary material available at 10.1186/s13098-022-00881-2.

## Introduction

Obesity is a complex, chronic and non-communicable disease, it has been recognized since 1998 by the world health organization as a disease because of its health and economic repercussions and its global impact. Obesity represents a major public health problem in many regions of the world, and concerns more than 35% of adults around the world. The Centers for Disease Control and Prevention reports that 53% of black women are obese. Due to the epidemiological and nutritional transition, developing countries have experienced a significant increase in the prevalence of obesity in recent decades. Obesity is characterized by an increase in adipose tissue along with an excess of fat mass and leads to a lot of co-morbidity and the risk of developing life-threatening metabolic complications such as type 2 diabetes. The risk of developing type 2 diabetes is thought to be 28 times higher in obese women compared to women with normal weight [1]. Most patients with type 2 diabetes are or have been obese, and the global epidemic of obesity largely explains the dramatic increase in the incidence and prevalence of type 2 diabetes in the world over these last decades [2].

Among the factors involved in the onset of type 2 diabetes in obese subjects, there are disorders of fatty acid metabolism and resistance to the action of insulin. Some authors believe that insulin resistance is the main culprit in the association between obesity and metabolic diseases such as type 2 diabetes [3]. It’s generally accepted that ‘abnormal’ insulin sensitivity precedes the clinical diagnosis of diabetes by up to 15 years [4]. Obesity is the major risk factor for insulin resistance in the general population [5] and it’s been observed that both increased abdominal subcutaneous and mostly perivisceral adipose tissue are strongly correlated with insulin resistance. Insulin resistance is a condition whereby insulin-induced glucose uptake is impaired in the insulin-sensitive tissue. The failure is a result of inhibition of the insulin signaling pathway. It’s widely believed that the availability and use of free fatty contribute to the development of skeletal muscle insulin resistance, as well as to increased liver glucose production [6]. Insulin resistance serves as a key link between obesity and type 2 diabetes [7], and understanding how obesity causes insulin resistance will improve our knowledge of type 2 diabetes and our ability to treat obesity-related complications type 2 diabetes.

The physiopathological mechanisms linking these two anomalies, injury fatty acid metabolism and insulin resistance, to the development of type 2 diabetes in the obese subject, involve an interaction between genetic and environmental factors maintained by the epigenetic mechanism. These interactions are complex and several genes are currently implicated in these interactions. Obesity pathogenesis involves an imbalance in energy metabolism that results from complex interactions between genetic, epigenetic, and environmental factors [8].

A cluster of differentiation 36 (CD36) is a transmembrane glycoprotein that belongs to class B scavenger receptors [9], present on the surface of many types of cells [9]. CD36 is involved in a wide variety of functions, including the regulation of energy metabolism through the potential binding of long-chain fatty acids [10] and elimination of oxidized plasma LDL cholesterol from macrophages and monocytes [11], fat storage, and differentiation of fat cells. A dysfunction of CD36 receptors leads to an imbalance in lipid metabolism [12], and insulin resistance [13]. CD36 is involved in the uptake of fatty acids, and several studies have indicated that there are relationships between elevated CD36 expression in different tissues and insulin resistance [14].

The first study which focused on the involvement of *CD36* single nucleotide polymorphism (SNP) in obesity was conducted by Pepino et al. [15]. Since then, several pioneering studies have reported similar results [16]. According to Bokor and al., the rs1527483 of the *CD36* gene has been associated with the onset of obesity by increasing the percentage of body fat and, consequently, the BMI [17]. The SNP rs1527483 is also implicated in the elevation of plasma levels of long-chain fatty acids in Caucasians [18]. Regarding the *CD36* rs3211867 polymorphism, their report is on an association of polymorphism with an increase in BMI and waist circumference (WC) with a risk for additional complications [19].

Some authors have suggested a decrease in the expression of *CD36* receptors in connection with methylation of the *CD36* gene [20].

To elucidate the association between the *CD36* gene polymorphisms and methylation with obesity and type 2 diabetes, we conducted this study whose general objective was to determine the interactions between *CD36* polymorphism (SNPs rs1761667, rs1527483, and rs3211867) and methylation of the *CD36* gene and their impacts on obesity and type 2 diabetes in Senegalese women. We hypothesize that the genetic and epigenetic changes at the *CD36* gene locus could influence the onset of obesity and type 2 diabetes in these women.

## Materials and methods

### Subjects and protocol

This case-control study was conducted in Senegal on female subjects. We initially enrolled a total of 50 healthy lean control females, 50 obese females, and 50 obese diabetic females. Subjects were recruited from the physiology laboratory in the faculty of medicine, pharmacy, and odontostomatology (FMPOS) of the University Cheikh Anta DIOP (UCAD) in Dakar, Senegal.

The inclusion criteria were older age 18 or more. The separation of lean and obese women was based on the World Health Organization (WHO) obesity diagnostic criteria set in 1998. On another hand, we recruited known and followed type 2 diabetic subjects according to the world health organization (WHO) diabetes diagnostic criteria set in 1979, justified in their medical follow-up file. We confirmed diabetic status using a pre-established questionnaire followed by clinical examinations, and tests on fasting blood glucose and glycosylated hemoglobin.

The three groups are matched according to the cases by age. All subjects in the lean control group were individuals free of diseases, extreme care was taken to exclude diseases that were determined by medical history and clinical and biological examinations. For the family history of control subjects, we found that 3 control subjects had at least one family member with lipid disorder, 8 control subjects had at least one obese family member, and 5 control subjects had at least one family member with type 2 diabetes. In order not to bias the study, all these control women were finally excluded. For the obese and obese diabetic subjects, the no inclusion criteria were as follows: pregnant or breastfeeding women and those with another pathological condition such as coronary artery disease, chronic liver pathology (determined by liver aspartate-amino transferase and alanine-amino transferase tests), and chronic kidney pathology (determined by measuring creatinine and urea). The obese had no other health problems besides obesity.

### Anthropometric and biologic measurements

At recruitment, all the subjects underwent an interview including demographic characteristics (age, sex, education level), medical histories (history of hypertension, duration of diabetes mellitus, use of lipid-lowering medications, anti-diabetes medications, and antiplatelet medications) through questionnaires. Clinical examination and anthropometric data (weight, height, waist circumference, hip circumference, waist-to-hip ratio, body mass index, body fat percentage, visceral fat level, and blood pressure) were collected from each subject (Additional files 1 and 2).

The biological samples have been taken the same day in the biochemistry laboratory in the FMPOS of UCAD. Samples were taken before the interviews after a 12-h overnight fast. The fasting venous blood was collected from all the participants at the fold of the elbow of the non-dominant arm. After total blood, serum and plasma were aliquoted and frozen at −80 °C for further analysis of blood parameters (Additional files 1 and 2).

We used an A25 biosystem device and reagents of the same brand to determine the concentrations of total cholesterol (Total-C), high-density lipoprotein cholesterol (HDL-C), triglycerides (TG), fasting blood glucose (glycemia), alanine aminotransferase (ALT), aspartate aminotransaminase (AST), urea and creatininemia by enzymatic methods. Low-density lipoprotein cholesterol (LDL-C) level was calculated by the Friedewald formula: LDL-C = Total C–HDL-C–TG/5.

A plasma sample was immediately used for glycosylated hemoglobin (HbA_1_c) determination by turbidimetric method (Architect i1000SR, Seattle, WA, USA).

Insulin was determined by enzyme-linked immunosorbent assay (ELISA) kits (Human Insulin ELISA Kit #A05322.96 wells, Version 0118, Bertin Bioreagent, France).

Insulin resistance was determined by the Homeostasis Model Assessment-Insulin Resistance (HOMA-IR) using the following Matthews formula: HOMA-IR = (insulin (mU/L) × glucose (mMol)/L))/22.5.

Levels of plasma sCD36 were measured by use of the commercially available Human CD36 enzyme-linked immunosorbent assay (ELISA) kits (CD36 (Human) ELISA Kit #KA4204, Version 09, Abnova, France).

Serum DNMT3a levels were measured by using the commercially available Human DNMT3a ELISA kits (Human DNA (cytosine-5)-methyltransferase 3a (DNMT3a) ELISA Kit KTE62548, Abbkine, Wuhan, Chine). In this study, we proceeded to the dosage of the enzyme DNMT3a to be able to support the methylation of the CD36 gene observed.

### Genotyping and methylation analysis

#### DNA extraction

Blood samples for DNA extraction were collected in EDTA K3 tubes. Genomic DNA (gDNA) was extracted from venous peripheral white blood cells using the commercially available Spin-column technique kit for DNA extraction from human whole blood (The PureLink® Genomic DNA Purification Mini Kit, Invitrogen™ by life technologies, CA K1820-02, Lot 1,977,075, Carlsbad, CA 92,008, USA) and the extracted DNA samples were stored at −20 °C for future use.

#### Determination of CD36 gene polymorphism

##### SNP selection for genotyping

In Table 1, we show some characteristics of the studied SNPs. To cover a good part of the genetic variability of the *CD36* gene in our study, we included 3 tagSNPs (rs3211867, rs1527483 et rs1761667). The criteria used in our SNP selection procedure were:

- minor allele frequency (MAF) > 0.05.
- 2 SNP blocks among the 5 large SNP blocks in the HapMap database of 2008: 1 tagSNP from block 4 (rs3211867, tagging 8 other SNPs) et 1 tagSNP from block 5 (rs1527483, tagging 1 other SNP).
- 1 tag SNP (rs1761667), which was not present in the HapMap database of 2008, but was chosen based on data from the literature.

**Table 1:** Characteristics of *CD36* gene SNPs investigated in the study

CD36 Variant	Alleles	refSNP ID	Location	Position
−31118	G > A	rs1761667	Exon 1A	Chromosome 7
11472	C > A	rs3211867	Intron 3	Chromosome 7
25444	G > A	rs1527483	Intron 11	Chromosome 7

The three tagSNPs were detected using standard assays on demand and using C_8314999_10 for rs1761667, C_8315330_10 for rs1527483, and C_1803793_10 for rs3211867.

Determination of *CD36* gene polymorphism was carried out with TaqMan® SNP Genotyping Assays (TaqPath™ ProAmp™ Master Mixes, ThermoFisher Scientific, MA, USA), by Real-Time polymerase chain reaction (RT-PCR) system and allele discrimination technique (TaqMan, Applied Biosystems, Assay Catalog number 4351379 Foster City, CA, USA) on a 96-well format and read by a StepOne Plus thermocycler (Applied Biosystems, Foster City, CA, USA). DNA was used at a final concentration of 20 ng in 4.5 μL. The PCR-mixture is composed of prepared DNA with distilled water (4.5 μL), 2X TaqMan® Master Mix (5 μL), and Stock de travail 20X Assay (0.5 μL) to reach a total volume of 10 μL. After an initial step (Pre-PCR Read: Holding Stage) of 30 s at 60 °C and 95 °C for 10 min to activate the AmpliTaq Gold, and UP, Enzyme Activation, the products were amplified (Cycling Stage) using 40 cycles of 15 s at 95 °C and 1 min 30 s at 60 °C, and the end for the Post-PCR Read (Holding Stage) 30 s at 60 °C. Then, allele detection and genotyping calling were performed using StepOne plus (Corbett Research, Mortlake, New South Wales, Australia) with the available installed software.

#### Determination of CD36 gene methylation

##### Sodium Bisulfite Modification

DNA was treated with sodium bisulfite using the Cp Genome™ Direct Prep bisulfite Modification Kit. In short, 500 ng of DNA in the protection buffer was mixed with 13 µl of 2× Extraction buffer, 1 µl of Proteinase K, and the rest was completed with RNase-free water. The final volume was 26 µl and it was incubated for 20 min at 50 °C, then it was centrifuged for 5 min for 10000×*g*. The supernatant was collected and mixed with the modification reconstitution reagent in PCR tubes. The conversion of bisulfite DNA was carried out in a thermocycler under the following conditions: 8 min at 98 °C, 3 h 30 min at 64 °C and a final step at 4 °C.

For purification, the samples were mixed with 600 µl of Binding buffer in spin columns and centrifuged while 30 s at 12000×*g*. Then the DNA was washed, 100 µl of wash buffer 1 then centrifuged while 30 s at 12000×*g*, 200 µl of wash buffer 2 then centrifuged while 30 s at 12000×*g* after 20 min of incubation and at the end 2 times with 200 µl of wash buffer 1 centrifuged while 30 s at 12000×*g*. The converted DNA was eluted with 10 µl of Elution buffer and stored at −20 °C.

##### Methylation-specific PCR (MS-PCR)

For the methylation analysis, we selected CpG islands located at −293.337 (promoter region) for *CD36*. Primers were designed with MethPrimer [21]. DNA was amplified with pairs of primers for each CpG island; one for the methylated template and the other for the unmethylated sequence, and PCR products were 103 bp. Primers for methylated and unmethylated sequences gave the same length of PCR products.

For PCR assay, a total volume of 25 µL containing the following: 2 µL of bisulfite-modified DNA was amplified in 12.5 µL PCR master mix (Platinum. Hot Start PCR Master Mix, Thermo Scientific Inc., USA), 0.5 µL of sense primer and 0.5 µL of antisense primer, 5 µL of CG enhancer (provided with Platinum. Master Mix), and 4.5 µL nuclease-free water.

PCR conditions were as follows: initial denaturation at 95 °C for 13 min, 40 cycles of 94 °C for 30 s, annealing for 30 min (Tm = 58 C), and 72 °C for 1 min for 35 cycles followed by a final extension step at 72 °C for 10 min.

For control, we used human methylated (positive control) and unmethylated (negative control) DNA furnished by the supplier (EpiTect PCR Control DNA Set, Qiagen, USA). In positive control, the pretreated DNA showed that the CpG was methylated and, in the same way, in negative control samples, all CpG were unmethylated. Amplification was done in the thermal cycler (iCycler C1000, Bio-Rad, Germany). Finally, 6 µL of PCR product was electrophoresed on 1% (w/v) agarose gel, containing ethidium bromide. The gels were visualized by ultraviolet light (Gel Doc imaging 2000, Bio-Rad).

### Statistical analysis

All variables were saved in an Excel table. Quantitative variables were described using mean ± standard deviation (SD) and qualitative variables using absolute values and percentages. Pairwise comparisons of the metabolic parameters between control, obese and obese-diabetic were evaluated by the unpaired student t-test and ANOVA test (post hoc test LSD). The Chi^2^ and Fisher test was used to compare the mean of the qualitative variables. Linear regression was used to assess the risk between sCD36 and the studied clinical and biochemical variables.

The results were considered significant when p ≤ 5%. The exploitation of the data was carried out by SPSS Statistics software version v23 × 64 (IBM, Chicago, IL, USA), and STATA version 12.0 (ISBN-13: 978-0-8400-6463-9).

## Results

### General and biochemical characteristics

The results of general, clinical, and biochemical data of participants according to the category are shown in Table 2. On the one hand, control subjects were different from the obese and obese diabetic subjects by their anthropometric parameters (waist size and BMI), their body composition (percent body fat and visceral fat level), but also by the parameters of carbohydrate and lipid metabolism (insulin, fasting blood glucose, glycated hemoglobin, and LDL cholesterol). It has shown us that obese subjects were different from obese diabetic subjects only by carbohydrate and lipid metabolism (fasting blood glucose, glycated hemoglobin, fasting insulin, HOMA-IR, and LDL cholesterol) which were obese-diabetic subjects.

**Table 2:** Comparison of clinical and biochemical characteristics between control, obese and obese diabetic groups

Variables	Control *n* = *50* *(a)*	Obese *n* = *50* *(b)*	Obese diabetic *n* = *50* *(c)*	*p*-value *(a) vs (b)*	*p*-value *(a) vs (c)*	*p*-value *(b) vs (c)*
Mean age (years)	49.98 ± 7.52	50.50 ± 8.76	51.06 ± 5.78	NS	NS	NS
Waist size (cm)	83.37 ± 9.28	102.28 ± 10.02	101.42 ± 11.66	< 0.0001	< 0.0001	NS
Height (cm)	166.53 ± 6.71	160.84 ± 7.71	162.70 ± 6.04	NS	NS	NS
Weight (kg)	67.48 ± 9.92	92.87 ± 14.22	87.98 ± 13.39	< 0.0001	< 0.0001	NS
BMI (kg/m^2^)	24.19 ± 2.74	34.30 ± 4.41	33.09 ± 4.30	< 0.0001	< 0.0001	NS
Percent body fat (%)	37.70 ± 6.39	46.08 ± 3.93	44.98 ± 4.56	< 0.0001	< 0.0001	NS
Visceral fat level	7.04 ± 1.76	11.54 ± 2.08	11.18 ± 2.31	< 0.0001	< 0.0001	NS
Glycated hemoglobin (%)	5.00 ± 1.76	5.57 ± 0.69	7.93 ± 2.07	0.047	< 0.0001	< 0.0001
Fasting blood glucose (g/L)	0.85 ± 0.13	0.85 ± 0.18	1.57 ± 0.78	NS	< 0.0001	< 0.0001
Fasting Insulin (µUI/mL)	17.53 ± 6.29	23.49 ± 7.45	31.45 ± 25.10	0.036	< 0.0001	0.01
HOMA-IR	3.66 ± 1.71	4.91 ± 2.01	12.19 ± 11.65	NS	0.002	0.02
Total Cholesterol (g/L)	2.13 ± 0.44	2.09 ± 0.41	2.27 ± 0.56	NS	NS	NS
HDL Cholesterol (g/L)	0.62 ± 0.14	0.58 ± 0.13	0.58 ± 0.16	NS	NS	NS
LDL Cholesterol (g/L)	1.40 ± 0.37	1.37 ± 0.40	1.64 ± 0.53	NS	0.004	0.009
Triglycerides (g/L)	0.80 ± 0.31	0.78 ± 0.33	0.86 ± 0.37	NS	NS	NS
ALT (UI/L)	17.57 ± 11.08	17.34 ± 8.88	19.68 ± 9.62	NS	NS	NS
AST (UI/L)	24.66 ± 1388	21.60 ± 8.00	24.16 ± 8.88	NS	NS	NS
Albuminemia (g/L)	44.76 ± 4.36	41.90 ± 4.85	42.82 ± 5.50	0.03	NS	NS
Bilirubinemia (g/L)	3.98 ± 2.19	5.54 ± 12.09	5.00 ± 3.49	NS	NS	NS
Creatinine (g/L)	8.87 ± 3.13	9.02 ± 2.79	10.59 ± 14.01	NS	NS	NS

*BMI* body mass index, *HOMA-IR* Homeostasis Model Assessment—Insulin Resistance, *ALT* alanine aminotransferase, *AST* aspartate aminotransferase

Besides, some parameters were significantly different between the three groups such as total cholesterol, HDL cholesterol, triglycerides, AST, ALT, and bilirubinemia. But that cannot err the results. See Table 2

### Allelic frequencies and genotypic distribution

In Table 3, Hardy–Weinberg Equilibrium (HWE_X_^2^) verifies that the genotype frequencies remain the same from generation to generation. A significant variation in the genotype frequencies was observed for rs1761667 among the obese subjects (HWE_X_^2^ = 5.56 p = 0.01), and for rs1527483 among the control subjects (HWE_X_^2^ = 21.54 p < 0.0001).

**Table 3:** Allele frequencies and genotype distributions of the *CD36* SNPs polymorphisms in each group of the population

SNP	Variables	Control *n* = *50*	Obese *n* = *50*	Obese diabetic *n* = *50*
*rs1761667*	HWE _X_^2^	0.59	5.56 (p = 0.01)	2.32
	* Genotyping number (%)*			
	GG	23 (46%)	21 (42%)	18 (36%)
	AA/AG	27 (54%)	29 (58%)	32 (64%)
	* Allele number (%)*			
	A	34 (34%)	30 (30%)	36 (36%)
	G	66 (66%)	70 (70%)	64 (64%)
	* A allele*			
	Control & Obese	OR = 0.83 [0.46–1.5] 95% IC		
	Control & Obese diabetic	OR = 1.09 [0.61–1.95] 95% IC		
*rs1527483*	HWE _X_^2^	21.54 (p = 0.001)	–	0.02
	* Genotyping number (%)*			
	GG	48 (96%)	50 (100%)	48 (96%)
	AA/AG	2 (4%)	–	2 (4%)
	* Allele number (%)*			
	A	3 (3%)	–	2 (2%)
	G	97 (97%)	100 (100%)	98 (98%)
	* A allele*			
	Control & Obese	OR = 0		
	Control & Obese diabetic	OR = 0.66 [0.11–4.04] 95% IC		
*rs3211867*	HWE _X_^2^	0.49	0.23	0.54
	* Genotyping number (%)*			
	CC	20 (40%)	23 (46%)	18 (36%)
	AA/AC	30 (60%)	27 (54%)	32 (64%)
	Allele *number (%)*			
	A	35 (35%)	31 (31%)	38 (38%)
	C	65 (65%)	69 (69%)	62 (62%)
	* A allele*			
	Control & Obese	OR = 0.83 [0.46–1.5] 95%IC		
	Control & Obese diabetic	OR = 1.14 [0.64–2.03] 95%IC		

*SNP* single nucleotide polymorphism, *OR* odds ratio, *HWE* Hardy–Weinberg Equilibrium

For rs1761667, rs1527483, and rs3211867, we found that they will have a protective or neutral effect on the occurrence of obesity and type 2 diabetes but without statistical significance.

### Variations in the characteristics of study group subjects depending on their genomic status

In Table 4, we found a statistically significant variation in certain clinical and biochemical parameters depending on the genotypic distribution of the *CD36* gene polymorphisms studied.

**Table 4:** Comparison of the clinical and biochemical data stratified by *CD36* gene polymorphisms in the control, obese and obese diabetic groups

Variables	Control *n* = *50*			Obese *n* = *50*			Obese diabetic *n* = *50*		
rs1761667	GG	AA/AG	*p* value	GG	AA/AG	*p* value	GG	AA/AG	*p* value
Body mass index (kg/m^2^)	24.58 ± 2.38	23.81 ± 3.05	NS	34.56 ± 4.14	34.14 ± 4.63	NS	34.24 ± 5.31	32.26 ± 3.25	NS
Waist size (cm)	84.00 ± 10.19	82.76 ± 8.47	NS	102.37 ± 9.86	102.23 ± 10.29	NS	104.95 ± 14.11	98.86 ± 8.91	0.04*
Percent body fat (%)	39.00 ± 4.94	36.46 ± 7.42	NS	46.39 ± 4.16	45.88 ± 3.85	NS	45.50 ± 4.61	44.25 ± 4.53	NS
Visceral fat level	7.29 ± 1.68	6.80 ± 1.83	NS	11.84 ± 2.04	11.35 ± 2.12	NS	11.71 ± 2.72	10.79 ± 1.92	NS
Glycated hemoglobin (%)	5.08 ± 0.57	4.93 ± 0.39	NS	5.50 ± 0.59	5.55 ± 0.73	NS	8.00 ± 2.01	7.81 ± 2.09	NS
HOMAIR	4.04 ± 1.64	3.32 ± 1.73	NS	5.20 ± 2.33	4.74 ± 1.81	NS	14.21 ± 13.15	10.42 ± 10.23	NS
Total cholesterol (g/l)	2.23 ± 0.42	2.04 ± 0.45	NS	2.12 ± 0.43	2.07 ± 0.40	NS	2.37 ± 0.65	2.19 ± 0.49	NS
HDL cholesterol (g/l)	0.63 ± 0.13	0.61 ± 0.14	NS	0.58 ± 0.16	0.58 ± 0.11	NS	0.64 ± 0.19	0.54 ± 0.12	0.04*
LDL cholesterol (g/l)	1.46 ± 0.30	1.34 ± 0.41	NS	1.38 ± 0.40	1.37 ± 0.41	NS	1.68 ± 0.60	1.62 ± 0.49	NS
Triglycerides (g/l)	0.84 ± 0.35	0.76 ± 0.28	NS	0.86 ± 0.43	0.73 ± 0.24	NS	0.84 ± 0.36	0.89 ± 0.39	NS
rs3211867	CC	AA/AC	*p* value	CC	AA/AC	*p* value	CC	AA/AC	*p* value
Body mass index (kg/m^2^)	23.46 ± 2.87	24.65 ± 2.59	NS	33.29 ± 3.50	35.32 ± 5.03	0.02*	31.94 ± 3.51	33.63 ± 4.58	NS
Waist size (cm)	80.95 ± 9.23	84.90 ± 9.13	NS	99.24 ± 8.72	105.32 ± 10.48	NS	100.0 ± 12.82	102.09 ± 11.21	NS
Percent body fat (%)	35.99 ± 7.94	38.78 ± 5.04	NS	45.38 ± 3.37	46.78 ± 4.38	NS	43.71 ± 5.17	45.28 ± 4.23	NS
Visceral fat level	6.74 ± 1.94	7.23 ± 1.63	NS	11.12 ± 1.79	11.96 ± 2.30	NS	10.19 ± 1.91	11.65 ± 2.36	NS
Glycated hemoglobin (%)	4.86 ± 0.44	5.09 ± 0.50	NS	5.52 ± 0.80	5.55 ± 0.53	NS	7.69 ± 1.92	7.98 ± 2.11	NS
HOMA-IR	4.01 ± 1.87	3.45 ± 1.60	NS	4.62 ± 1.96	5.21 ± 2.05	NS	11.86 ± 10.96	12.39 ± 10.15	NS
Total cholesterol (g/l)	2.11 ± 0.48	2.15 ± 0.43	NS	2.00 ± 0.42	2.18 ± 0.39	NS	2.03 ± 0.38	2.37 ± 0.60	0.03*
HDL cholesterol (g/l)	0.59 ± 0.15	0.64 ± 0.13	NS	0.55 ± 0.12	0.61 ± 0.13	NS	0.54 ± 0.17	0.61 ± 0.16	NS
LDL cholesterol (g/l)	1.45 ± 0.37	1.37 ± 0.37	NS	1.35 ± 0.41	1.40 ± 0.39	NS	1.44 ± 0.38	1.73 ± 0.57	NS
Triglycerides (g/l)	0.73 ± 0.25	0.84 ± 0.34	NS	0.71 ± 0.28	0.85 ± 0.37	NS	0.88 ± 0.44	0.86 ± 0.34	NS
rs1527483	GG	AA/AG	*p* value	GG	AA/AG	*p* value	GG	AA/AG	*p* value
Body mass index (kg/m^2^)	24.44 ± 2.51	18.75 ± 1.48	0.003**	34.24 ± 4.44	–	–	33.25 ± 4.33	31.65 ± 1.90	NS
Waist size (cm)	83.38 ± 9.14	75.00 ± 4.24	NS	102.76 ± 9.68	–	–	101.73 ± 11.69	97.50 ± 10.61	NS
Percent body fat (%)	37.96 ± 6.37	36.60 ± 1.27	NS	45.78 ± 4.22	–	–	44.75 ± 4.65	44.30 ± 1.13	NS
Visceral fat level	6.98 ± 1.68	6.00 ± 1.41	NS	11.53 ± 2.09	–	–	11.27 ± 2.32	9.50 ± 0.71	NS
Glycated hemoglobin (%)	5.00 ± 0.50	4.90 ± 0.14	NS	5.50 ± 0.57	–	–	7.88 ± 2.07	8.65 ± 0.49	NS
HOMA-IR	3.66 ± 1.83	2.95 ± 0.78	NS	4.82 ± 2.12	–	–	7.83 ± 11.12	7.42 ± 10.50	NS
Total cholesterol (g/l)	2.11 ± 0.44	2.49 ± 0.88	NS	2.11 ± 0.39	–	–	2.25 ± 0.55	2.42 ± 1.03	NS
HDL cholesterol (g/l)	0.62 ± 0.13	0.75 ± 0.16	NS	0.58 ± 0.13	–	–	0.58 ± 0.16	0.55 ± 0.15	NS
LDL cholesterol (g/l)	1.36 ± 0.37	1.87 ± 0.33	NS	1.40 ± 0.38	–	–	1.63 ± 0.54	1.80 ± 0.48	NS
Triglycerides (g/l)	0.81 ± 0.31	0.62 ± 1.56	NS	0.78 ± 0.34	–	–	0.85 ± 0.35	1.18 ± 0.71	NS

*HOMA-IR* Homeostasis Model Assessment-Insulin Resistance

^*^p ≤ 0.05

**p ≤ 0.01

***p ≤ 0.0001

For rs1761667, only obese-diabetic subjects had statistically significant different parameters depending on the genotypic distribution, these were waist size (p = 0.04) and HDL-cholesterol (p = 0.04) which are significantly higher in subjects of the GG genotype.

For rs3211867, obese subjects harboring the AA/AC genotype had a significantly higher BMI (p = 0.02) and total cholesterol (p = 0.03) than obese subjects harboring the CC genotype. At the same time, the obese diabetic subjects harboring the AA/AC genotype have a total cholesterol level significantly higher than the obese diabetic subjects harboring the CC genotype (p = 0.03).

For rs1527483, only the control subjects in the GG genotype had a significantly higher BMI than the control subjects harboring the AA + AG genotype (p = 0.003).

Tables 3, 4 show the allelic frequencies and genotype distributions of three *CD36* gene polymorphisms in control, obese and obese diabetic subjects. Subjects harboring the minor A allele of *CD36* rs1527483 are rare so we didnot calculate the mean of their parameters.

### Association between SNPs and the clinic and para clinic parameters

In Table 5, we studied the relations between the SNPs *CD36* gene polymorphisms and the studied parameters in the different subject groups.

**Table 5:** Association between clinical and biochemical parameters with the *CD36* gene methylation

SNPs	Indicators	Control *n* = *50*	Obese *n* = *50*	Obese diabetic *n* = *50*
		Odds Ratio 95% IC; *p* value	Odds Ratio 95% IC; *p* value	Odds Ratio 95%IC; *p* value
rs1761667	Body mass index (0/1)	0.09 [0.64–1.75]; 0.82	1.31 [0.99–1.74]; 0.06	0.78 [0.34–1.82]; 0.57
	Waist size (0/1)	0.90 [0.72–1.13]; 0.37	0.80 [0.65–0.98]; 0.03*	0.81 [0.60–1.09]; 0.17
	Percent body fat (0/1)	0.94 [0.70–1.24]; 0.65	1.41 [0.94–2.12]; 0.09	0.70 [0.36–1.35]; 0.28
	Visceral fat level (0/1)	1.54 [0.65–3.65]; 0.33	0.62 [0.34–1.14]; 0.12	3.44 [0.45–26.60]; 0.24
	HbA_1_c (0/1)	0.32 [0.04–2.57]; 0.28	1.26 [0.35–4.57]; 0.72	0.67 [0.35–1.29]; 0.23
	IR-HOMA (0/1)	0.71 [0.40–1.26]; 0.24	0.82 [0.53–1.27]; 0.38	0.95 [0.87–1.05]; 0.34
	sCD36	1.00 [0.99–1.00]; 0.56	1.00 [0.99–1.00]; 0.64	1.00 [0.99–1.00]; 0.37
	Dnmt3a	1.05 [0.99–1.11]; 0.11	1.01 [0.98–1.03]; 0.61	0.96 [0.89–1.02]; 0.19
	Total cholesterol (0/1)	0.05 [0.00–59.94]; 0.41	0.13 [0.00–105.51]; 0.56	0.00 [0.00–2.98]; 0.09
	HDL cholesterol (0/1)	0.22 [0.00–14.01]; 0.47	1.14 [0.01–100.15]; 0.91	0.02 [0.00–1.06]; 0.048*
	LDL cholesterol (0/1)	0.40 [0.08–1.96]; 0.26	0.91 [0.21–3.89]; 0.52	0.81 [0.28–2.36]; 0.70
	Triglycerides (0/1)	0.40 [0.06–2.53]; 0.33	0.29 [0.04–1.93]; 0.20	4.45 [0.30–6.93]; 0.64
rs3211867	Body mass index (0/1)	1.58 [0.90–2.78]; 0.11	1.12 [0.86–1.45]; 0.83	0.62 [0.32–1.20]; 0.16
	Waist size (0/1)	0.92 [0.71–1.18]; 0.49	0.11 [0.93–1.31]; 0.25	0.99 [0.75–1.31]; 0.94
	Percent body fat (0/1)	1.11 [0.84–1.46]; 0.46	0.80 [0.54–1.19]; 0.27	0.97 [0.55–1.72]; 0.92
	Visceral fat level (0/1)	0.76 [0.30–1.89]; 0.55	0.97 [0.52–1.81]; 0.92	3.72 [0.90–15.33]; 0.07
	HbA_1_c (0/1)	4.93 [0.29–84.02]; 0.27	0.80 [0.25–2.57]; 0.71	1.97 [0.75–5.15]; 0.17
	IR-HOMA (0/1)	0.41 [0.19–0.91]; 0.03*	1.12 [0.77–1.62]; 0.55	0.98 [0.90–1.07]; 0.63
	sCD36	0.99 [0.99–1.00]; 0.62	1.00 [0.99–1.00]; 0.75	1.00 [0.99–1.00]; 0.39
	Dnmt3a	0.99 [0.94–1.03]; 0.55	1.01 [0.98–1.03]; 0.65	0.97 [0.90–1.04]; 0.35
	Total cholesterol (0/1)	0.16 [0.00–877.66]; 0.67	0.55 [0.00–506.55]; 0.86	0.21 [0.00–2.98]; 0.09
	HDL cholesterol (0/1)	14.29 [0.18–1115.17]; 0.23	56.21 [0.50–6279.58]; 0.09	21.01 [0.25–1718.01]; 0.17
	LDL cholesterol (0/1)	0.56 [0.11–2.75]; 0.47	1.36 [0.33–5.62]; 0.66	3.66 [0.85–15.84]; 0.08
	Triglycerides (0/1)	2.39 [0.01–58.43]; 0.59	13.80 [0.31–605.36]; 0.17	1.40 [0.02–92.94]; 0.23
rs1527483	Body mass index (0/1)	0.33 [0.08–1.26]; 0.11	–	0.89 [0.56–1.42]; 0.62
	Waist size (0/1)	–	–	–
	Percent body fat (0/1)	–	–	–
	Visceral fat level (0/1)	0.69 [0.30–1.60]; 0.39	–	0.53 [0.17–1.64]; 0.27
	HbA_1_c (0/1)	–	–	–
	IR-HOMA (0/1)	0.67 [0.21–2.14]; 0.50	–	1.02 [0.88–1.17]; 0.82
	sCD36	–	–	–
	Dnmt3a	0.90 [0.75–1.07]; 0.23	–	0.66 [0.32–1.37]; 0.26
	Total cholesterol (0/1)	–	–	–
	HD cholesterol (0/1)	6.49^e+3^ [0.00–8.70^e+9^]; 0.22	–	0.26 [0.00–3.99^e+3^]; 0.79
	LDL cholesterol (0/1)	–	–	–
	Triglycerides (0/1)	0.08 [0.00–31.72]; 0.41	–	7.56 [0.24–238.49]; 0.25

*HOMA-IR* Homeostasis Model Assessment-Insulin Resistance, *sCD36* Soluble CD36 protein, *DNMT3a* DNA (Cytosine-5-)-Methyltransferase 3 Alpha, 0/1: 0 (normal), 1 (abnormal or hight)

*: p ≤ 0.05

**: p ≤ 0.01

***: p ≤ 0.0001

The A allele of rs1761667 would be associated with a decrease in waist size in the obese group [OR = 0.80 (0.65–0.98); 0.03] and with a decrease in HDL-cholesterol in the obese diabetic group [OR = 0.02 (0.00–1.06); 0.048].

In the control subjects, we noted that the A allele of rs3211867 would be associated with a decrease in HOMA-IR [OR = 0.41 (0.19–0.91); 0.03].

### Methylation of CpG islands of the CD36 promoter

*CD36* CpG islands in the promoter of the *CD36* gene were significantly more methylated in obese and obese-diabetic than in control women.

**Figure 1:**
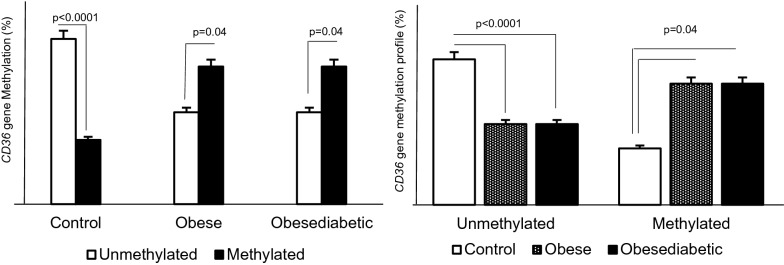
Relationships between methylation, obesity, and type 2 diabetes. In Panel A, the symbol without filling is unmethylated, and the symbol with a single filling is methylated. In Panel B, the symbol without filling is control; the symbol with the filling pattern is obese and the symbol with a single filling is obese diabetic. *p* value ≤ 5% was considered significant.

According to the risk calculations, we found that obese women are 1.36 times more likely to have *CD36* gene methylation than control healthy women [RR = 1.36; OR = 1.96 (0.96—3.81); p = 0.04]. If we add the two groups, obese and obese diabetic subjects, after the Chi2 test they will be 3.86 times more exposed to *CD36* gene methylation than the lean control. We found the same percentage of methylated subjects in the obese and obese diabetics. See the Fig. 1.

### Variations in anthro-biological parameters according to CD36 gene methylation

From Table 6, we see that obese diabetics with *CD36* gene methylation have a significantly higher LDL-cholesterol level than obese diabetics without *CD36* gene methylation (p = 0.01).

**Table 6:** Comparison of the clinical and biochemical data stratified by *CD36* gene methylation in the control, obese and obese diabetic groups

Variables	Control			Obese			Obese diabetic		
	Unmethylated	Methylated	*p* value	Unmethylated	Methylated	*p* value	Unmethylated	Methylated	*p* value
Body mass index (kg/m^2^)	24.31 ± 2.64	23.94 ± 2.97	NS	34.97 ± 5.51	33.69 ± 3.59	NS	32.63 ± 3.27	33.78 ± 4.95	NS
Waist size (cm)	83.44 ± 8.94	83.21 ± 9.97	NS	103.05 ± 9.03	102.52 ± 10.13	NS	102.86 ± 10.71	100.56 ± 12.63	NS
Percent body fat (%)	38.34 ± 6.42	36.23 ± 6.04	NS	45.54 ± 4.55	45.82 ± 4.04	NS	44.39 ± 4.71	45.39 ± 4.71	NS
Visceral fat level	7.12 ± 1.59	6.50 ± 1.83	NS	11.53 ± 2.09	11.50 ± 2.15	NS	10.81 ± 1.97	11.56 ± 2.59	NS
Fasting blood glucose (g/l)	0.88 ± 0.12	0.80 ± 0.12	NS	0.88 ± 0.24	0.87 ± 0.26	NS	1.74 ± 0.82	1.45 ± 0.74	NS
Glycated hemoglobin (%)	5.03 ± 0.57	4.92 ± 0.68	NS	5.50 ± 0.70	5.61 ± 0.70	NS	8.31 ± 2.13	7.64 ± 2.02	NS
IR-HOMA	3.80 ± 1.85	3.22 ± 1.64	NS	5.23 ± 2.33	4.57 ± 1.93	NS	9.28 ± 11.65	7.06 ± 10.88	NS
Total cholesterol (g/l)	2.10 ± 0.46	2.19 ± 0.48	NS	2.07 ± 0.35	2.14 ± 0.44	NS	2.14 ± 0.50	2.35 ± 0.60	NS
HDL cholesterol (g/l)	0.62 ± 0.14	0.63 ± 0.13	NS	0.57 ± 0.15	0.60 ± 0.13	NS	0.57 ± 0.17	0.58 ± 0.15	NS
LDL cholesterol (g/l)	1.35 ± 0.37	1.45 ± 0.41	NS	1.38 ± 0.31	1.42 ± 0.43	NS	1.55 ± 0.39	1.71 ± 0.62	0.01**
Triglycerides (g/l)	0.86 ± 0.31	0.65 ± 0.25	NS	0.82 ± 0.31	0.75 ± 0.35	NS	0.83 ± 0.43	0.89 ± 0.33	NS

*HOMA-IR* Homeostasis Model Assessment-Insulin Resistance

^*^: p ≤ 0.05

**: p ≤ 0.01

***: p ≤ 0.0001

**Figure 2:**
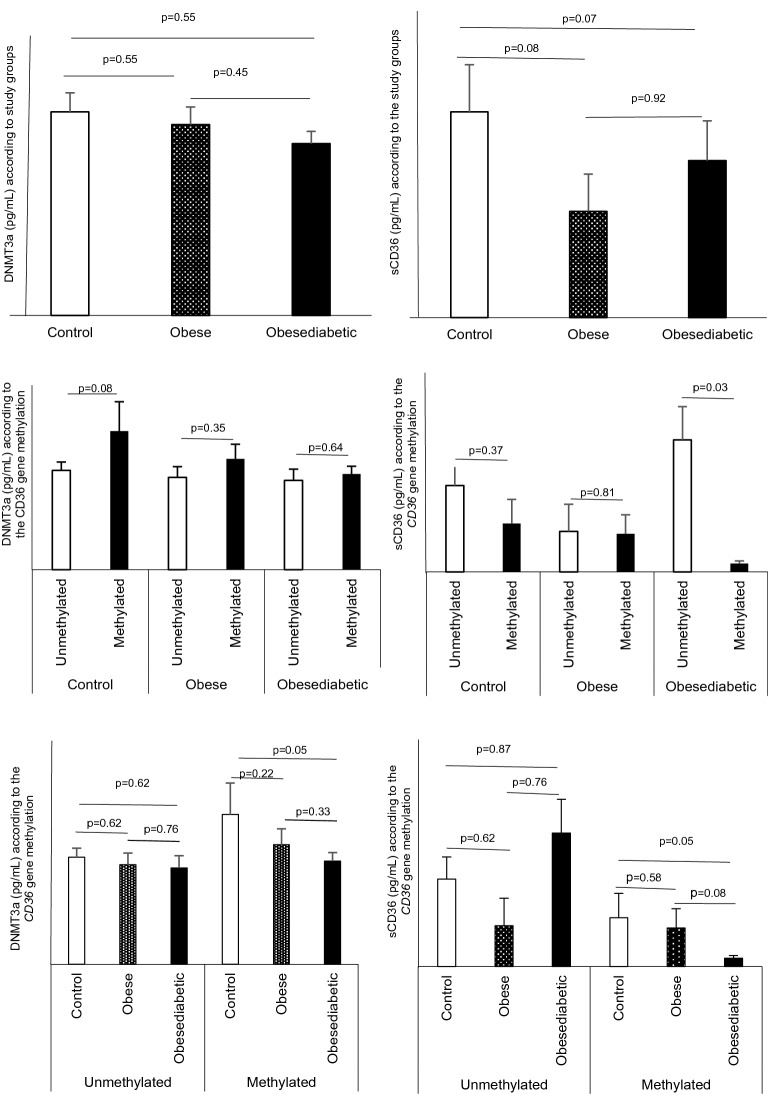
Variations in DNMT3a and sCD36 levels according to the CD36 gene methylation. In Panels A - B - E - F, the symbol without filling is control, the symbol with the filling pattern is obese and the symbol with single filling is obese diabetics. In Panel C and D, the symbol without filling is unmethylated, and the symbol with the single filling is methylated. p value ≤ 5% was considered significant.

From Fig. 2, the sCD36 level was reduced in the obese group and obese diabetic group compared to the control subject but not significantly. We see that, in each group, the sCD36 level was reduced in the subjects with *CD36* gene methylation compared to the subjects without *CD36* gene methylation and this was statistically significant in the obese diabetic group (p = 0.03). At the same time, we found that the sCD36 level was significantly reduced in the obese diabetic subjects with *CD36* gene methylation compared to the control subjects with *CD36* gene methylation (p = 0.05).

In the same Fig. 2, the DNMT3a level was higher in control with *CD36* gene methylation compared to control with unmethylated *CD36* gene. Control subjects whose *CD36* gene was methylated had remarkably more DNMT3a compared to obese diabetics with *CD36* gene methylation (p = 0.05).

### Association between CD36 gene methylation and the other studied clinical-biologic parameters

In Table 7, we found that the *CD36* gene methylation would be associated with a decrease in the percentage of total body fat in the obese diabetic subjects [0.42 (0.18–0.94); 0.04].

**Table 7:** Risk of variations in clinical and biochemical data stratified by *CD36* gene methylation in the control, obese and obese diabetic groups

Variables	Control * n* = *50*	Obese * n* = *50*	Obese diabetic * n* = *50*
	Odds Ratio [95% IC]; *p* value	Odds Ratio [95% IC]; *p* value	Odds Ratio [95% IC]; *p* value
Body mass index (0/1)	1.48 [0.83–2.65]; 0.18	0.89 [0.72–1.11]; 0.31	1.36 [0.55–3.36]; 0.51
Waist size (0/1)	0.99 [0.85–1.15]; 0.88	0.99 [0.91–1.08]; 0.83	0.96 [0.79–1.78]; 0.72
Percent body fat (0/1)	0.99 [0.70–1.40]; 0.94	0.99 [0.73–1.35]; 0.95	0.42 [0.18–0.94]; 0.04*
Visceral fat level	0.25 [0.05–1.15]; 0.08	1.12 [0.64–1.96]; 0.68	4.06 [0.75–21.99]; 0.10
Glycated hemoglobin (0/1)	4.40 [0.17–113.49]; 0.37	1.13 [0.41–3.09]; 0.82	0.28 [0.07–1.20]; 0.09
HOMA-IR (0/1)	1.06 [0.45 -2.49]; 0.90	0.88 [0.63–1.23]; 0.46	0.95 [0.86–1.05]; 0.29
sCD36 (0/1)	1.00 [0.99–1.00]; 0.96	1.00 [0.99–1.00]; 0.12	1.00 [0.99–1.00]; 0.35
DNMT3a (0/1)	1.01 [0.99–1.04]; 0.24	1.00 [0.98–1.03]; 0.69	0.99 [0.96–1.02]; 0.59
Total cholesterol (0/1)	2.53 [2.58–11.11]; 0.22	3.59 [0.01–1748.98]; 0.91	2.33 [0.79–6.89]; 0.13
HDL cholesterol (0/1)	3.18 [0.03–349.79]; 0.63	0.45 [0.00–5592.54]; 0.87	3.04 [0.08–108.87]; 0.54
LDL cholesterol (0/1)	3.49 [0.55–22.01]; 0.18	0.35 [0.00–74.44]; 0.70	2.02 [0.64–6.34]; 0.23
Triglycerides (0/1)	0.09 [0.01–1.90]; 0.07	0.28 [0.00–8.29]; 0.46	1.75 [0.36–8.42]; 0.48

*HOMA-IR* Homeostasis Model Assessment-Insulin Resistance, *sCD36* Soluble CD36 protein, *DNMT3a* DNA (Cytosine-5-)-Methyltransferase 3 Alpha, 0/1: 0 (normal), 1 (abnormal or hight)

*p ≤ 0.05

**: p ≤ 0.01

***p ≤ 0.0001

### Association between polymorphisms and methylation of the CD36 gene

We found that the rs3211867 polymorphism was significantly associated with *CD36* gene methylation in the control and obese diabetic groups (respectively p = 0.049; p = 0.002). However, the SNP 1527483 has no relationship noted with *CD36* gene methylation. See the Table 8

**Table 8:** Associations between polymorphism and methylation of the *CD36* gene

Variables	Control *n* = *50*		Obese *n* = *50*		Obese diabetic *n* = *50*	
Methylation	Unmethylated	Methylated	Unmethylated	Unmethylated	Unmethylated	Methylated
SNPs						
rs1761667						
GG	16 (66.67)	8 (33.33)	9 (47.37)	10 (52.63)	6 (28.57)	15 (71.43)
AA/AG	21 (80.77)	5 (19.23)	9 (29.03)	22 (70.97)	16 (55.17)	13 (44.83)
Total	37 (74.00)	13 (26.00)	18 (36.00)	32 (64.00)	22 (44.00)	28 (56.00)
*p* value	0.34		0.23		0.09	
rs3211867						
CC	17 (89.47)	2 (10.53)	8 (32.00)	17 (68.00)	12 (75.00)	4 (25.00)
AA/AC	20 (64.52)	11 (35.48)	10 (40.00)	15 (60.00)	10 (29.41)	24 (70.59)
Total	37 (74.00)	13 (26.00)	18 (36.00)	32 (64.00)	22 (44.00)	28 (56.00)
*p* value	0.049*		0.56		0.002**	
rs1527483						
GG	35 (72.92)	13 (27.08)	18 (36.00)	32 (64.00)	20 (41.67)	28 (58.33)
AA/AG	2 (100)	–	–	–	2 (100)	–
Total	37 (74.00)	13 (26.00)	18 (36.00)	32 (64.00)	22 (44.00)	28 (56.00)
*p* value	0.54		–		0.19	

*SNPs* single nucleotide polymorphisms

*p ≤ 0.05

**p ≤ 0.01

***p ≤ 0.0001

### Impact of sCD36 levels on the clinical and biochemical parameters in control, obese and obese diabetic groups

Table 9 shows that in the control group, an increase in sCD36 levels would be associated with a decrease in total cholesterol and triglyceride levels but would be associated with an increase in LDL cholesterol levels. For the obese group, an increase in sCD36 levels would be associated with an increase in fasting insulin levels and a decrease in glycated hemoglobin levels. An increase in the sCD36 levels would be associated with an increase in the triglyceride levels in the obese diabetic group.

**Table 9:** relations between sCD36 levels and the variety of clinical and biochemical parameters

sCD36 (pg/ml)	Control *n* = 50			Obese *n* = 50			Obese diabetic *n* = 50		
	Coeff	Std. Err	*p* value	Coeff	Std. Err	*p* value	Coeff	Std. Err	*p* value
Body mass index (kg/m^2^)	176.81	233.80	0.46	74.81	114.17	0.52	−481.37	715.51	0.51
Waist size (cm)	−67.18	94.07	0.48	−97.24	77.34	0.22	−79.88	326.75	0.81
Percent body fat (%)	23.96	108.41	0.83	−187.99	163.12	0.26	−671.33	588.52	0.27
Visceral fat level	−586.59	370.94	0.12	296.47	253.73	0.25	2367.23	1556.68	0.15
Fasting insulin (µUI/ml)	−291.92	453.63	0.52	490.99	205.91	0.02*	68.46	325.67	0.84
Fasting glucose (g/l)	−3642.67	10184.42	0.72	9044.59	6624.70	0.18	−192.41	4725.54	0.97
Glycated hemoglobin (%)	−545.70	886.09	0.54	−1196.26	513.01	0.03*	−748.46	761.10	0.34
HOMA-IR	1329.10	2095.85	0.53	−1693.51	992.31	0.10	−33.75	751.58	0.97
Dnmt3a (pg/ml)	− 5.60	18.29	0.76	−6.23	10.82	0.57	−59.39	66.36	0.38
Total cholesterol (g/l)	−7647.56	2861.04	0.01*	−1297.12	2948.87	0.66	1818.41	7018.14	0.11
HDL cholesterol (g/l)	−3986.54	5026.10	0.43	−3869.63	4370.21	0.38	15727.13	12665.98	0.23
LDL cholesterol (g/l)	11,547.28	2963.45	< 0.0001*	1278.08	2556.97	0.62	9638.22	6619.96	0.17
Triglycerides (g/l)	−2528.50	1237.69	0.048*	−850.91	1598.50	0.06	9937.41	3666.00	0.02*

*BMI* body mass index, *HOMA-IR* Homeostasis Model Assessment-Insulin Resistance, *sCD36* soluble CD36 protein, *DNMT3a* DNA (Cytosine-5-)-Methyltransferase 3 Alpha

*p ≤ 0.05

**p ≤ 0.01

***p ≤ 0.0001

## Discussion

During the last decade, the genetic and especially epigenetic relationships between obesity and type 2 diabetes mellitus have attracted increasing interest. This interest stems, on the one hand, from the galloping expansion of obesity and consequently of type 2 diabetes mellitus and, on the other hand, from the epigenetic regulation of gene expression often influenced by environmental stimuli. As a result, using molecular biology methods, several genetic and epigenetic changes associated with obesity have been identified [22]. The *CD36* role in obesity and its related complication type 2 diabetes prompted us to investigate its singles nucleotides polymorphisms and gene methylation association with obesity and obese-diabetic in Senegalese women.

We have noted, in this present study, variations in the frequencies and distribution of the *CD36* allele and genotype.

For rs1761667, the frequencies and distribution of the *CD36* allele and genotype were not significantly different between the groups (control, obese and obese diabetic). However, the AA/AG genotype of the promoter polymorphism rs1761667 in the *CD36* gene was more prevalent in all groups (control (54%), obese (58%), and obese diabetic (64%)).

In the obese group, the A allele of rs1761776 would be significantly a protective factor against an increase in waist size [OR = 0.80 (0.65–0.98); 0.03]. In addition, subjects harboring GG genotype had a higher waist size compared to subjects harboring the AA/AG genotype (0.04). At the same time, the A allele of rs1761667 would be associated with a decrease in the blood level of HDL cholesterol in obese diabetics [OR = 0.02 (0.00–1.06); 0.049]. Moreover, obese diabetics carrying the GG genotype had a higher blood level of HDL cholesterol than carriers of the AA/AG genotype (p = 0.04). Allele A of rs1761667 would protect against abdominal obesity in obese subjects, but it would be associated with a decrease in the HDL cholesterol blood level. The A allele wasn't associated with a risk of obesity, on the contrary, would protect against abdominal obesity in already obese subjects, which doesn’t corroborate the data in the literature. According to Love-Gregory et al., the A allele of SNP rs1761667 is associated with an increase in fat intake and causes a decrease in the expression and function of the CD36 proteins, which can lead to a feeling of the fattiness of lower intensity and therefore exposure to obesity [23]. An association was found between genotypes of the *CD36* promoter rs1761667 and the metabolic profile in our obese diabetic subjects, which is in agreement with previous reports. Authors have already shown an influence of the rs1761667 polymorphism on the lipid profile, GG genotype would be associated with a decrease in the blood level of free fatty acids. [18].

For rs3211867, the effects observed would vary according to the groups in the sample. The obese subjects harboring AA/AC genotype had significantly higher BMI (p = 0.02) and total cholesterol (p = 0.02) than obese subjects harboring the CC genotype. At the same time, obese diabetics harboring the AA/AC genotype had higher total cholesterol levels compared to obese diabetics harboring the CC genotype (p = 0.03). Our study showed that the AA/AC genotype of a promoter polymorphism of *CD36* rs3211867 increases exposure to obesity and hypercholesterolemia. This observation is supported by the study of Bokor et al. [17] which reported that *CD36* rs3211867 was associated with a higher risk of obesity.

We found that the rs1527483 polymorphism of the *CD36* gene is rare in our study population, which means that the variant A allele is not very present in the study population. The control subjects harboring the GG genotype of rs1527483 had significantly higher BMI compared to the control harboring the AA/GG genotype of rs1527483 (p = 0.03). It’s already been proven by many authors in other populations that rs1527483 was significantly associated with a greater risk of obesity [17]. Only in our study carriers of the GG genotype would be more exposed to obesity than carriers of the AA/AG genotype, this would be contrary to the data of the literature. According to previous investigations, regarding lipid profile, no significant difference was found with different *CD36* rs1527483 genotypes, neither within the same group nor between the different groups. However, another study conducted by Ma et al. demonstrated that *CD36* rs1527483 is implicated in high no esterified plasma fatty acids (NEFA) levels [18].

The study of epigenetics and its involvement in metabolic diseases is still a young research field. Several studies have investigated genome-wide DNA methylation and its association with obesity and type 2 diabetes.

In our present study, we found that *CD36* gene methylation was more remarkable in obese and obese-diabetic subjects compared to control subjects but it is not significant [RR = 1.36; OR = 1.96 (0.96–3.81; p = 0.04)]. *CD36* CpG islands in the promoter of the *CD36* gene were significantly more methylated in obese and obese-diabetic than in control females. If we add the two groups of obese and obese diabetic subjects, both will be 3.86 times more associated with *CD36* gene methylation than the control subjects. Similar results were shown by Xu et al. who reported in their study that the variance of DNA methylation was greater in the obese cases than in the lean control and differential methylation could predict obesity with around 70% confidence [24]. In the same way, some authors showed that the variance of DNA methylation was greater in the obese cases than in the healthy normal-weight cases [25]. Methylation represents a form of epigenetic memory that persists into adulthood and may play a role in the developmental programming of obesity. Thus, the DNA methylation status of a particular gene may regulate the risk of obesity for life. The DNMT3a enzyme is a member of the family and it preferentially targets unmethylated DNA and provides de novo methylations. The methylation of DNA is possible thanks to enzymes of the gene family of DNA methyltransferases (DNMTs) whose role is to affix a methyl group on the cytosines of DNA [26].

Likewise, Charlotte’s study said DNA methylation may cause several pathologies and contribute to obesity and type 2 diabetes [25]. Importantly, the authors went further saying that DNA methylation is associated with obesity and predicted future risk of type 2 diabetes, which is a major clinical condition associated with obesity. The DNMT3a enzyme is a member of the family and it preferentially targets unmethylated DNA and provides de novo methylations. The methylation of DNA is possible thanks to enzymes of the gene family of DNA methyltransferases (DNMTs) whose role is to affix a methyl group on the cytosines of DNA [26].

Obese diabetic subjects with *CD36* gene methylation had significantly fewer plasma sCD36 (p = 0.03) and more LDL-cholesterol (p = 0.01) than obese-diabetic subjects without *CD36* gene methylation. The level of sCD36 is reduced in the obese group and obese diabetic group compared to the control subject but not significantly. We see that, in each group, the sCD36 level is reduced in the subjects with *CD36* gene methylation compared to the subjects without *CD36* gene methylation and this is statistically significant in the obese diabetic group (p = 0.03). At the same time, we found that the sCD36 level is significantly reduced in the obese diabetic subjects with *CD36* gene methylation compared to the control subjects with *CD36* gene methylation (p = 0.05).

At the same time, the study shows that in the control group, an increase in sCD36 levels would decrease the risk of an increase in total cholesterol and triglycerides levels but would increase the risk of an increase in LDL cholesterol levels. For the obese group, an increase in sCD36 levels would decrease the risk of an increase in HbA_1_c levels but would increase the risk of an increase in fasting insulin levels. An increase in the sCD36 levels would increase the risk of an increase in the triglycerides level in the obese diabetic group. The sCD36 encoded by the *CD36* gene is involved in macrophage cholesterol and phospholipids transport. Studies have already reported an increase in circulating cholesterol by partial or complete *CD36* deficiency [27], especially LDL cholesterol. Hypermethylation of the *CD36* gene reduces mRNA and therefore decreases the level of sCD36, which leads to high serum lipid levels such as LDL cholesterol.

We found that the rs3211867 polymorphism was significantly associated with *CD36* gene methylation in the control and obese diabetic groups (respectively p = 0.05; p = 0.002). However, the SNP 1527483 has no relationship noted with *CD36* gene methylation.

Our results will be in perfect agreement with those of Love-Gregory et al. where it is that the SNPs are also associated with DNA methylation sites that are related to reduced *CD36* mRNA and higher serum lipids, but mixed-model analyses indicated that the SNPs and methylation independently influence *CD36* mRNA [28]. Changes in *CD36* DNA methylation influence both *CD36* expression and lipids [28].

In the present study, we determined the effects of common *CD36* variants and *CD36* gene methylation on obesity and its related complication type 2 diabetes.

The differences reported in these findings as well as other previous studies may be the interactions of SNPs with other variants in *CD36* gene or gene–gene interaction, genetic heterogeneity, ethnicity of the population, the small sample size and made up only of women, differences in the genetic constitution and environmental properties, gene-environment interactions in various populations.

Several limits should be noted from this study. We chose an exclusively female sample because in our country the prevalence of obesity is higher among women and is particularly worrying because of the high risk of developing metabolic disorders such as insulin resistance and type 2 diabetes. In addition, the sample size is relatively small. However, we intend to expand the study on a larger scale involving both men and women.

## Conclusion

The present study indicated that both differential SNP variability and differential methylation could predict obesity and its related complications to the type 2 diabetes. It results from an intricate interaction between genetic make-up and the environment, suggesting it’s orchestrated by epigenetic mechanisms. This present study presented the first evidence which suggests that *CD36* plays an important role in lipid metabolism and *CD36* gene polymorphisms and methylation might be a factor predisposing to obesity and its related complication type 2 diabetes in a Senegalese population. Importantly, the study’s results highlight the potential utility of assessing *CD36* expression and its common SNP genotypes. Therefore, implementing strategies for the early identification and prevention of obesity is fundamental to tackling this pandemic disease and its co-morbidities, which are the leading causes of death worldwide. These findings need confirmation further by more conclusive, and prospective studies incorporating both sexes, and a wide range of ethnicities with a gene expression and functional assay in the future.

## Supplementary Information

**Additional file 1.** Sample of the Data Collection Sheet on Control Subjects. Paragraph I: sociodemographic characteristics Paragraph II: history and terrain + way of life. This part talks about the personal and family defects of each control subject and their medical history. It also talks about the subject's lifestyle as a sedentary, smoking, alcoholic subject. Paragraph III: clinical characteristics such as the data of their anthropometry, their body composition, and their cardiovascular constant. Paragraph IV: biological parameters, this part corresponds to the laboratory data, namely the lipid and carbohydrate parameters, and renal function.

**Additional file 2.** Sample of the Data Collection Sheet on Diabetes Subjects. Paragraph I: sociodemographic characteristics Paragraph II: history and terrain + way of life. This part talks about the personal and family defects of each control subject and their medical history. It also talks about the subject's lifestyle as a sedentary, smoking, alcoholic subject. Paragraph III: clinical characteristics such as the data of their anthropometry, their body composition, and their cardiovascular constant. This part speaks in addition to the history of the diabetes disease such as the date of onset, the circumstances of discovery, the current treatment, and the presence or absence of diabetes complications. Paragraph IV: biological parameters, this part corresponds to the laboratory data, namely the lipid and carbohydrate parameters, and renal function.

## Data Availability

All data generated or analyzed during this study are included in this published article and its supplementary information files.
