# Supplementary material for: Association between polymorphisms and hypermethylation of CD36 gene in obese and obese diabetic Senegalese females
Source: Diabetol Metab Syndr. 2022 Aug 18;14:117. doi: 10.1186/s13098-022-00881-2 (PMC9386198; doi:10.1186/s13098-022-00881-2)
Supplement: Supplementary file 2 — Additional file 2. Sample of the Data Collection Sheet on Diabetes Subjects. Paragraph I: sociodemographic characteristics Paragraph II: history and terrain + way of life. This part talks about the personal and family defects of each control subject and their medical history. It also talks about the subject's lifestyle as a sedentary, smoking, alcoholic subject. Paragraph III: clinical characteristics such as the data of their anthropometry, their body composition, and their cardiovascular constant. This part speaks in addition to the history of the diabetes disease such as the date of onset, the circumstances of discovery, the current treatment, and the presence or absence of diabetes complications. Paragraph IV: biological parameters, this part corresponds to the laboratory data, namely the lipid and carbohydrate parameters, and renal function. [file 13098_2022_881_MOESM2_ESM.docx]

**DATA COLLECTION SHEET FOR SUBJECTS WITH TYPE 2 DIABETES ALL FEMALE**

Survey date ………………………………

Patient Index……………………………………. Order number………………………….

Date of collection………………………………… Tel……………………………………….

Address…………………………………………….

**I – Sociodemographic characteristics**

First names………………………………… Last name…………………………

Age:…………. years Ethnicity………………….............

**Occupation :**

Civil servant □ Self-employed □ Volunteer □ Housewife □

Unemployed □ Retired □ Student □ Other □

**Schooling**: Yes □ No □

Level of study language of study…………………………………………………

**II-History and terrain + way of life**

**1-Personal**

** Medical:**

Other illness: no □ yes □

Which one: Hypertension □ Obesity □ Dyslipidemia □

Heart disease □ which one…………………………………………………...

Others: ………………………………………………………………………

** Surgical:**  yes no 

Type of surgery:…………………………………………………………………………..

 **Gyneco-obstetrics**

If the woman is with pregnancy in progress………………………………………………………..

Others:……………………………………………………………………………………..

**2- family history**

Hypertension  Diabetes Obesity  Dyslipidemia 

Heart disease: no  yes  which one…………………………………

Others:………………………………………………………………………….

**3 - Lifestyle**

Smoking: yes □………….. no □

If yes number of packages per day:……….…………………………………..

Duration of smoking:…………………………………………………………

If weaned give the date of weaning: ……………………………………………..

Alcohol: yes □………… no □

Sedentary: yes □ no □

Physical activity: yes □…………… no □ Frequency per week:……………………..

**II- Clinical Characteristics**

**1. Constants**

Height:……..m / Weight:………kg / Waist size:………cm / hip circumference:…… cm

BMI:………… kg /m² / Fat mass:…….% / Visceral fat:

SBP:……………… mmHg / DBP:…………….. mmHg / Heart rate:……………. Bpm

**2. Known type 2 diabetes**

Age of onset:……….years Duration of evolution:…………years

Complications: no  yes  which one…………………………………

**3. Treatment**

Diet: tracked  not tracked 

Antidiabetics: biguanides  SH  Glinides  Insulin  IAG  IDDP-4 

Other associations :

Antihypertensives: ACI  ARA2  IC  BB  Diuretics 

Traditherapy: yes  no ****

**III- Biological parameters**

In the laboratory

**Lipid profile**

Total cholesterol:………..g/l, HDL cholesterol:…………g/l, LDL cholesterol:…………g/l, Triglycerides :………….g/l

Blood sugar:.…………g/l, HbA1c:………….%, Calcemia:………. g/l

**Prélèvement sang total pour génotypage**
